# Optimization of Biodegradation Characteristics of *Sphingopyxis* sp. YF1 against Crude Microcystin-LR Using Response Surface Methodology

**DOI:** 10.3390/toxins14040240

**Published:** 2022-03-27

**Authors:** Isaac Yaw Massey, Tangjian Peng, Cai Danping, Fei Yang

**Affiliations:** 1 Hunan Provincial Key Laboratory of Clinical Epidemiology, Xiangya School of Public Health, Central South University, Changsha 410017, China; mriymassey@csu.edu.cn; 2 Hunan Province Key Laboratory of Typical Environmental Pollution and Health Hazards, School of Public Health, University of South China, Hengyang 421009, China; pengtangjian@usc.edu.cn (T.P.); 20202014210994@stu.usc.edu.cn (C.D.); 3 Laboratory of Ecological Environment and Critical Human Diseases Prevention of Hunan Province, School of Basic Medical Sciences, Hengyang Medical School, University of South China, Hengyang 421009, China

**Keywords:** MC-LR, *Sphingopyxis* sp. YF1, biodegradation, multiple environmental factor optimization, response surface methodology

## Abstract

*Sphingopyxis* sp. YF1 has proven to be efficient in biodegrading microcystin (MC)-leucine (L) and arginine (R) (MC-LR); however, the optimal environmental factors to biodegrade the toxin have not been investigated. In this study, the biodegrading characteristics of *strain* YF1 against MC-LR were assessed under diverse environmental factors, including temperature (20, 30 or 40 °C), pH (5, 7 or 9) and MC-LR concentration (1, 3 or 5 µg/mL). Data obtained from the single-factor experiment indicated that MC-LR biodegradation by strain YF1 was temperature-, pH- and MC-LR-concentration-dependent, and the maximal biodegradation rate occurred at 5 µg/mL/h. Proposing Box-Behnken Design in response surface methodology, the influence of the three environmental factors on the biodegradation efficiency of MC-LR using strain YF1 was determined. A 17-run experiment was generated and carried out, including five replications performed at the center point. The ANOVA analysis demonstrated that the model was significant, and the model prediction of MC-LR biodegradation was also validated with the experimental data. The quadratic statistical model was established to predict the interactive effects of the environmental factors on MC-LR biodegradation efficiency and to optimize the controlling parameters. The optimal conditions for MC-LR biodegradation were observed at 30 °C, pH 7 and 3 µg/mL MC-LR, with a biodegradation efficiency of 100% after 60 min. The determination of the optimal environmental factors will help to unveil the detailed biodegradation mechanism of MC-LR by strain YF1 and to apply it into the practice of eliminating MC-LR from the environment.

## 1. Introduction

The intensification of agriculture and rapid development of industries have increased eutrophication in various waterways worldwide. It is of interest that natural factors and global climate change are also contributing factors as water temperatures rise and severe droughts occur [1,2]. The widespread occurrence of cyanobacterial blooms may deteriorate to affect water sources, including drinking water and recreational uses. This may subsequently induce detrimental consequences on aquatic ecosystems and human health, as many cyanobacteria can generate an array of toxic metabolites, including microcystins (MCs), the most commonly detected cyanobacterial toxin [1,3,4].

MCs are a group of monocyclic heptapeptide hepatotoxins that are generated by species of cyanobacteria, including *Microcystis*, *Aphanizomenon*, *Anabaena*, *Cylindrospermopsis*, *Nostoc* and *Oscillatoria*. Approximately 279 variants of MC have been characterized across the globe [5,6]. Among the many MC variants identified, MC-leucine (L) and arginine (R) (MC-LR) appear to be the most frequently detected, very toxic and well-studied variants worldwide. MCs share a general cyclic structure cyclo-(-D-Ala-L-Leu-D-MeAsp-L-Arg-Adda-D-Glu-Mdha), where Adda is (2S, 3S, 8S, 9S) 3-amino-9 methoxy-2, 6, 8-trimethyl-10-phenyldeca-4, 6-dienoic acid; D-MeAsp is D-erythro-b-methylaspartic acid; Mdha is N-methyldehydroalanine and X and Z are variable L-amino acids [7,8,9].

It is well established that the toxin predominantly occurs in freshwater, marine habitats and desert environments. The increasing water hazards caused by this toxin have globally been documented [1,3]. In Caruaru, Brazil, MCs were attributed to the 131 hemodialysis patient casualties [10]. MCs affected more than 2,000,000 residents around Lake Taihu for days without drinking water, eventually leading to the tap water crisis [4]. Moreover, the presence of MCs in drinking water has been associated with the high incidence of liver cancer among the populace in China, Florida (USA) and Serbia [11].

MC-LR exerts immense toxic harm on humans, animals and plants. Exposure to this toxin has demonstrated tissues and organs damage in various mammals [3,12,13,14]. Toxicity of MC-LR occurs through the inhibition of protein phosphatases 1 (PP1) and PP2A, and it can also induce oxidative stress [14,15,16]. Due to the numerous concerns about the potential acute and chronic toxicity of MC-LR, the International Agency for Research on Cancer classified this toxin as a group 2B carcinogen [17], and the World Health Organization (WHO) suggests 1 µg/L and a maximum of 20,000 cyanobacterial cells mL^−1^ or 10 µg/L of chlorophyll-a (where about 2–4 µg/L of MCs is expected) as the toxin’s guidelines for drinking water quality and a safe recreational water environment, respectively [18,19]. The guidelines stipulated by WHO imply that the MC-LR concentration in drinking and recreational water should not be higher than that. However, in polluted environments, where MC-LR concentration is much higher that what is being stipulated, adverse health hazards may occur when humans and animals are exposed to the toxin, hence the need for treatment.

Providing safe drinking water to humans and animals is a public health concern. Although a number of methods have been proposed to remove MC-LR from water, the exploitation of bacteria to biodegrade this toxin represents a promising biological method, owing to the safety and low-cost compared to the other methods [20,21,22]. Concerning the biological degradation of MC, a number of bacteria capable of biodegrading the toxin have been isolated under different conditions from various waterways [5,7,23]. Besides, several researchers have also studied the biological degradation of MCs under various environmental factors, including temperature, pH and toxin concentration [8,9,24]. Interestingly, majority of the studies utilized the univariate method, where one parameter is altered while the rest remain constant. Zhang et al. [25] noted that this method did not reflect the synergistic effects among process parameters. The application of response surface method (RSM), which incorporates a new statistical design tool, is gradually gaining recognition for assessing multivariable influence. The RSM has widely been utilized in many process optimization controls [26,27,28,29]; however, only little evidence exists in literature regarding the process-optimizing control for MC-LR degradation [25,30]. Zhang et al. [25] found that the optimum conditions for MC-LR degradation were indicated at a gas–liquid interface gap of 10 mm, pulsed voltage of 16 kV and oxygen flow rate of 120 L/h via a gas–liquid hybrid discharge system. Guo et al. [30] also recently reported 30 °C, pH 7 and 2 µg/mL MC-LR as the optimal MC-LR biodegradation following RSM application to evaluate the influence of varying environmental factors using bacterial community YFMCD4. The evidence suggests that RSM can be an economic and reasonable experimental design to conduct a comprehensive study on biological degradation of MC-LR under different environmental conditions.

In our previous study, a novel indigenous bacterium, *Sphingopyxis* sp. YF1, with a high efficient capacity for MC-degradation was successfully acquired from Lake Taihu [7]. MC-LR was sequentially biodegraded into a final potential product CO_2_. Using mass spectrometer and multi-omics analyses, specific proteins, including microcystinase, linearized-microcystinase, tetrapeptidease, PAAase, PaaA, PaaG and PaaZ, were responsible for the MC-LR biodegradation into linearized MC-LR, tetrapeptide, Adda, phenylacetic acid, phenyl-CoA, acetyl-CoA and then CO_2_ via the classic tricarboxylic cycle [7]. Strain YF1 has been proven to be efficient in the biodegradation of MC-LR, but its optimal environmental factors for MC-LR biodegradation have not been studied. Besides, RSM has not been used to optimize the biodegrading conditions of MC-LR using a bacterial strain. The objectives of the present study were (1) to assess the biodegrading characteristics of strain YF1 under diverse environmental factors, including temperature, pH and MC-LR concentration, with respect to the bacterial biodegradation capacity and (2) to optimize the MC-biodegrading characteristics of strain YF1 via RSM. The Box-Behnken Design (BBD) was proposed in RSM to assess the influence of the environmental factors on the MC-LR biodegradation efficiency. Analysis of variance (ANOVA) was also used to ascertain the validity of the model. The illumination of the optimal environmental factors will help us to reveal the detailed MC-LR biodegradation mechanism by strain YF1 and to apply it into the practice of removing MC-LR from the environment.

## 2. Results and discussion

### 2.1. Crude MC-LR Biodegrading Characteristics under Varying Environmental Factors Using Strain YF1

**Figure 1:**
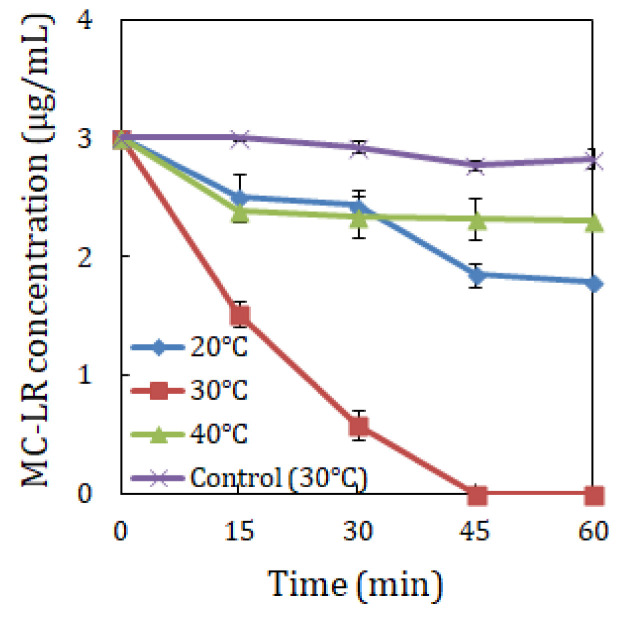
Effect of temperature on crude MC-LR biodegradation by *Sphingopyxis* sp. YF1. The error bars demonstrate the standard deviation of two replicates.

**Figure 2:**
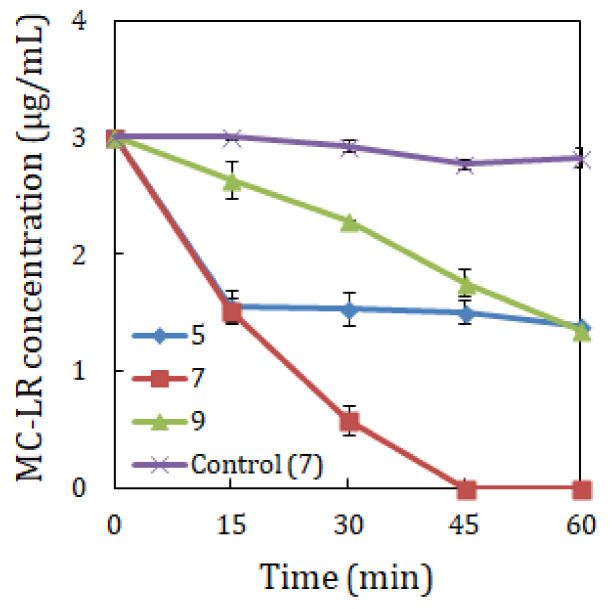
Effect of pH on crude MC-LR biodegradation by *Sphingopyxis* sp. YF1. The error bars indicate the standard deviation of two replicates.

**Figure 3:**
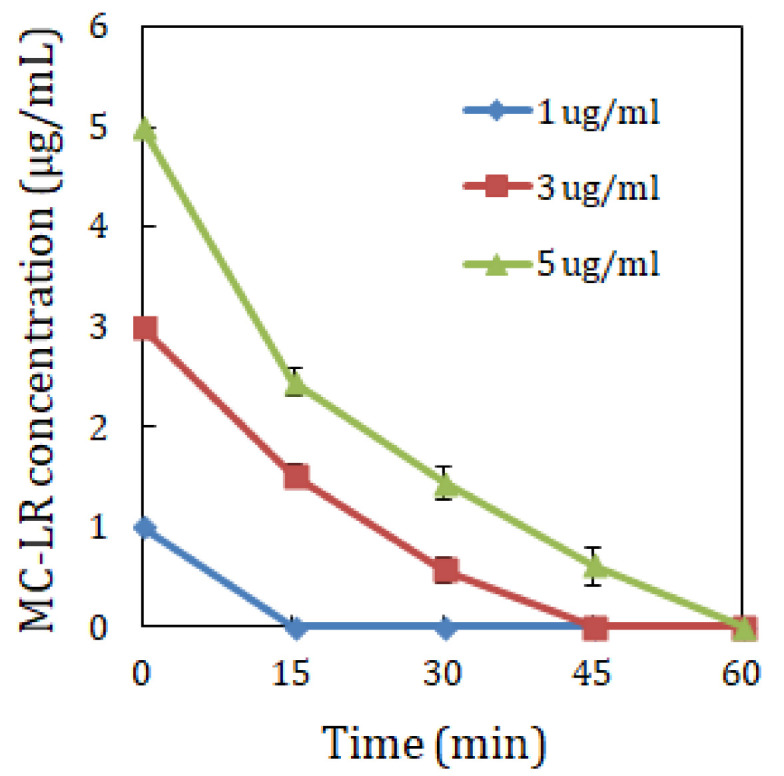
Effect of crude MC-LR concentrations on the biodegradation of crude MC-LR by *Sphingopyxis* sp. YF1. The error bars uncover the standard deviation of two replicates.

MC-LR biodegradation using strain YF1 was assessed under different incubation temperatures, pH values and MC-LR concentrations. The findings as shown in Figure 1, Figure 2 and Figure 3 uncovered that MC-LR biodegradation rates were influenced by the varying environmental factors.

Figure 1 demonstrated that at pH 7, strain YF1 biodegraded 3 µg/mL MC-LR at a rate of 0.020, 0.067 and 0.012 µg/mL/min at 20 °C, 30 °C and 40 °C, respectively, in 60 min. Data in Figure 2 indicated that at 30 °C, strain YF1 biodegraded 3 µg/mL MC-LR at a rate of 0.027, 0.067 and 0.028 µg/mL/min at pH 5, 7 and 9, respectively, in 60 min. Data in Figure 3 uncovered that at pH 7 and 30°C, strain YF1 biodegraded 1, 3 and 5 µg/mL MC-LR at a rate of 0.067, 0.067 and 0.083 µg/mL/min, respectively, in 60 min. The findings revealed that 0.083 µg/mL/min was the highest biodegradation rate by strain YF1, which occurred at pH 7, 30 °C and 5 µg/mL MC-LR. It was observed that without strain YF1, MC-LR biodegradation did not take place in the control samples.

Microbial biodegradation is regarded as an eco-friendly, effective and efficient strategy to remove MC-LR from natural waters without generating any harmful metabolites. In this study, the efficient and novel MC-biodegrading strain YF1 obtained from Lake Taihu was found to biodegrade MC-LR, and it exhibited a maximal toxin biodegradation rate at 5 µg/mL/h. Compared with earlier studies, the biodegrading rate of strain YF1 was much greater than that of *Stenotrophomonas acidaminiphila* MC-LTH2, *Paucibacter* sp. CH, *Rhizobium* sp. TH, *Ochrobactrum* sp. FDT5 and *Sphingopyxis* sp. a7 [8,23,24,31,32]. which were reported as remarkable microorganisms from natural water bodies with efficient capacities for MC-biodegradation. It is of interest that the diverse conditions to examine the MC-LR biodegradation ability, including different media, types of MC (crude extract or standard/pure), bacteria concentrations and other chemicals as well as physical conditions used in the laboratory, may account for the differences in the biodegradation rates of MC-LR. 

Researchers have demonstrated that varying temperatures, pH and MC-LR concentrations play a vital role in the MC-LR biodegradation process [5,9,24,33]. In the current study, these environmental factors were found to significantly influence the MC-LR biodegrading rates using strain YF1, and the highest MC-LR biodegradation conditions were observed at pH 7, 30 °C and 5 µg/mL MC-LR. Temperature has been reported to be a major factor which influences the toxin’s biodegradation. Studies indicate that a temperature between 11 °C and 30 °C may be effective to biodegrade MCs, where more rapid biodegradation in most cases occurs at higher temperatures [5,34,35,36]. Park et al. [34] showed that MC-LR, MC-RR and MC-YR decomposition using *Sphingomonas* sp. Y2 was very slow from 5 °C, 10 °C and to 20 °C, and the optimal decomposition rate was recorded at 30 °C. Wang et al. [35] noted that at 22 °C, 25 °C and 30 °C, the highest MC-LR biodegradation rate was obtained. However a reduced MC-LR biodegradation rate was observed at temperatures below 22 °C and above 30 °C. Utilizing a bacterial consortium, Ramani et al. [36] also indicated that MC-LR elimination was poor at 28 °C, and the maximum elimination was attained at 24 °C and 26 °C. In the present study, temperature was shown to play a fundamental role in the microbial biodegradation, with the highest biodegradation rate at a maximum temperature and a rapid decline in the biodegradation rate observed resulting from a decrease or an increase in temperature. At 3 µg/mL MC-LR biodegradation by strain YF1 under diverse incubation temperatures of 20 °C, 30 °C and 40 °C (Figure 1), the highest biodegradation rate was documented at 30 °C. At this temperature, thorough MC-LR biodegradation was found after 45 min. The findings disclosed that at 40 °C, MC-LR biodegradation was very slow. Even though MC-LR biodegradation was also slow at 20 °C, it was moderately quicker than that at 40 °C; conversely, total toxin biodegradation was not revealed. The data signify that the biodegradation of MC-LR is strongly dependent upon temperature. The slow, poor or no removal of MC-LR discovered in certain incubated temperatures suggests that the temperatures may have inactivated the MC-LR biodegrading bacteria.

pH, a measure of the acidity or alkalinity of a solution, is an important physical factor for MCs biodegradation. For this reason, it was essential to assess the impact of pH on the MC-LR biodegradation, as the pH of numerous waterways differs throughout the cyanobacterial blooms period and becomes extremely alkaline [33]. Various researchers have demonstrated that MCs biodegradation was strongly dependent on diverse pH levels. For instance, Okano et al. [33] reported that MC-LR and MC-RR removal using *Sphingopyxis* sp. C-1 occurred at different pH levels, including pH 11 and pH 10. The greatest MC removal activity of strain C-1 was between pH 8.45 and pH 6.52, and the optimum MC removal was obtained at pH 7. Fujimoto et al. [37] also found that *Sphingopyxis* sp. MG‒15 and *Novosphingobium* sp. MG‒22 could biodegrade MC-LR, MC-RR and MC-YR under a strong alkaline pH 10 condition. In the current investigation, pH was also indicated to play an essential function in the microbial biodegradation, with the highest biodegradation rate at a neutral pH and a rapid decline or increase in the biodegradation rate observed resulting from a decrease or an increase in pH. At 3 µg/mL MC-LR biodegradation by strain YF1 under varying pH levels of 5, 7 and 9 (Figure 2) also realized the highest biodegradation rate at pH 7. At this pH level (neutral pH 7), total MC-LR biodegradation was discovered after 45 min. It was revealed that at pH 5 (a strong acidic level), MC-LR biodegradation was very slow. Although MC-LR biodegradation was also slow at pH 9 (weak alkaline level), it was to some extent faster than that at pH 5; however, total toxin biodegradation was not documented. The observed results imply that biodegradation of MC-LR was strongly dependent upon pH. 

The concentration of MC-LR has also been documented to influence the toxin’s biodegradation. MC-LR biodegradation rate is said to increase when the MC-LR concentration decreases. When the MC-LR concentration increases, the toxin’s biodegradation rate is said to decline [5,9,24,37]. MC-LR biodegradation under different toxin concentrations of 1, 3 and 5 µg/mL by strain YF1 (Figure 3) realized the highest biodegradation rate at 5 µg/mL MC-LR, 30 °C and pH 7. At this toxin concentration, total MC-LR biodegradation was found after 60 min, and the biodegradation took place at an increasing rate. Although at 1 µg/mL MC-LR (at 30 °C and pH 7) and 3 µg/mL MC-LR (at 30 °C and pH 7), total biodegradation was established after 15 and 45 min, respectively, the biodegradation occurred at a decreasing rate. Thus, the observed experimental biodegradation of MC-LR progressed in a comparable way within 60 min, and the optimal MC-LR biodegradation was found at 30 °C, pH 7 and 5 µg/mL MC-LR at a biodegradation rate of 5 µg/mL/h using strain YF1.

### 2.2. Response Surface Methodology for Environmental Factor Selection and Analysis

Biodegradation of MC-LR is mostly influenced by the presence of some environmental factors [5,9,24]. Based on the conducted MC-LR biodegradation experiment using strain YF1, the interacting environmental factors (temperature, pH and MC-LR concentration) were further investigated via the established response surface model. The real levels and coded levels of each independent environmental factor are indicated in Table 1. Using RSM, these three influencing environmental factors were analyzed according to BBD. MC-LR biodegradation under varying test environmental factors in a 17-run experiment using the Design expert (Design-Expert 8.0.6) software is shown in Table 2. The temperature, pH and MC-LR concentration varied, and the MC-LR 60 min biodegradation rate was between 29.7% and 100%. Table 3 illustrates the results of the ANOVA analysis and statistical significance levels of the three influencing environmental factors investigated. The obtained results were used in verifying the statistical significance of the model for 60 min biodegradation of MC-LR using strain YF1.

The model’s F-value of 68.68, as found in Table 3, indicates that the model was significant, as the *p*-value of <0.0001 is less than 0.05. This implies there was only a 0.01% chance that a model F-value this large could have occurred as a result of noise. The effects of the first order (X2) and second order (X1^2^, X2^2^ and X3^2^) were highly significant. However, X1 and X3 and the interactive environmental factors (X1X2, X1X3 and X2X3) were insignificant (Table 3), since their *p*-values were greater than 0.05. The most significant environmental factor observed to influence MC-LR biodegradation was pH, with a *p*-value of 0.0007, F-value of 33.48 and a mean square of 649.80.

**Figure 4:**
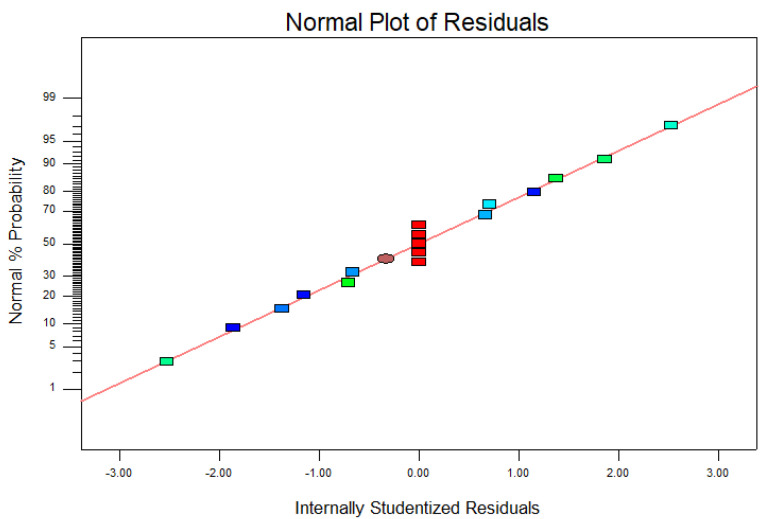
The normal plot of residual graph displaying a normal distribution. By displaying a normal distribution, it confirms the normality assumptions made earlier and the independence of the residuals.

Liu et al. [38] reported that the residual’s information, such as lack of fit, is a crucial aspect and can be utilized to assess the model’s adequacy. Wang et al. [39] also found that the normal plot of residual graph is a vital way of establishing the systematic deviations of the claim that errors are normally dispersed and that error divergence is alike. In this study, the normal distribution observed in the normal plot of the residual graph (Figure 4) was pleasing. This implies and further confirms that the residuals were independent, with a normality assumption. Using the lack of fit to confirm the model’s adequacy, the selected quadratic model was sufficient to explain the data, given that the pure error of zero demonstrates that the lack of fit was not significant.

The “Pred R−Squared” of 0.8208 was in reasonable agreement with the “Adj R−Squared” of 0.9744. “Adeq Precision” measures the signal to noise ratio. A ratio greater than 4 is desirable. The ratio of 21.173 indicates an adequate signal. This model can be used to navigate the design space. Using the low coefficient of variation (C.V% = 7.17), the regression was also verified. The R−Squared of 0.9888 and Adj R−Squared of 0.9744 of the model given agreed well with the actual experiment, indicating a significant relationship between the independent environmental factors and the response value. Consequently, it could be used for the theoretical prediction of MC-LR biodegradation over 60 min utilizing strain YF1.

By fitting the coefficient estimate of the environmental factors via RSM based on the BBD as depicted in Table S1, the final equation in terms of the coded environmental factors to show MC-LR biodegradation utilizing strain YF1 was given as

Y = 100.00 − 0.89 (X1) − 9.01 (X2) + 1.35 (X3) − 2.73 (X1X2) + 5.15 (X1X3) − 0.95 (X2X3) − 35.79 (X1^2^) − 17.79 (X2^2^) − 28.36 (X3^2^)

and the final equation in terms of actual environmental factors was also given as

Y = −482.04375 + 21.56500 (X1) + 62.55000 (X2) + 37.15625 (X3) − 0.13625 (X1X2) + 0.25750 (X1X3) − 0.23750 (X2X3) − 0.35788 (X1^2^) − 4.44688 (X2^2^) − 7.09062 (X3^2^)

Taking together, the single factor experimental and response surface model (statistical) results affirm the remarkable influence of the three environmental factors examined in this study on the biodegradation of MC-LR using strain YF1. The response surface model was made as an effective way of reflecting the MC-LR biodegrading procedure influenced by the controlling environmental factors. Utilizing RSM to determine the optimal conditions for MC-LR biodegradation by strain YF1, the ANOVA analysis revealed that the model was significant and that the model’s prediction of MC-LR biodegradation was also validated and fit with the experiment. Considering the biodegradation efficiency of MC-LR in the range of 29.7% and 100%, pH 7, 30 °C and 3 µg/mL MC-LR emerged as the optimal MC-LR biodegradation conditions, yielding a biodegradation efficiency of MC-LR of 100%. The two replicated single factor experimental test results also unveiled that the highest MC-LR biodegradation (5 µg/mL) was realized at the optimal environmental factors of pH 7 and 30 °C using strain YF1. The data confirmed the validity of the experimental and statistical strategies. The observed findings may prove that RSM is indeed an applicable method in optimizing MC-LR biodegrading environmental factors using strain YF1. The successful utilization and reliability of RSM in this study should be employed for further studies to optimize the environmental conditions for the biodegradation of MCs using a bacterial strain.

### 2.3. Interactive Effects of Multiple Environmental Factors on Crude MC-LR Biodegradation by Strain YF1

**Figure 5:**
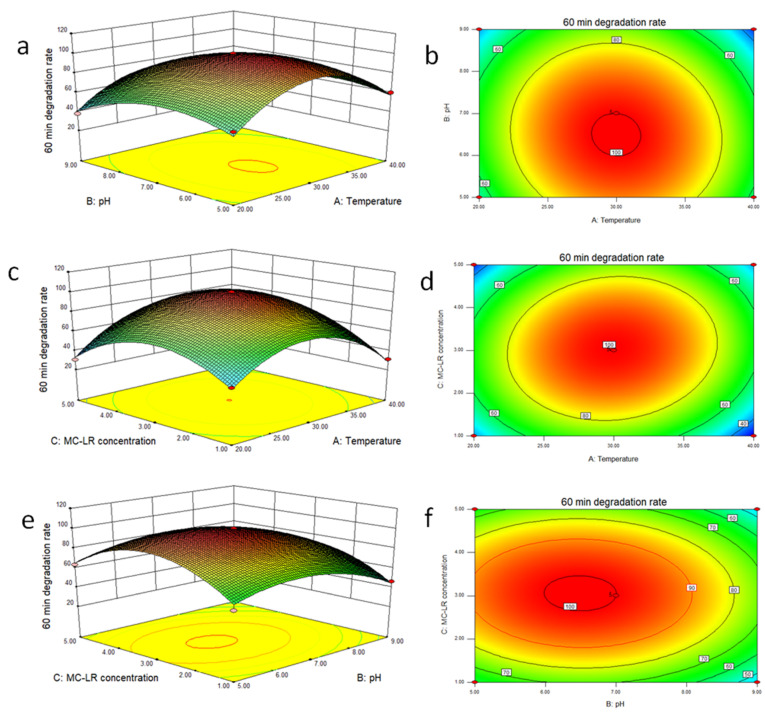
Response surface of environmental factors ((**a**) Response surface of temperature and pH on crude MC-LR biodegradation ratio, (**b**) Contour map showing the influence of temperature and pH on crude MC-LR biodegradation ratio, (**c**) Response surface of crude MC-LR concentration and temperature on crude MC-LR biodegradation ratio, (**d**) Contour map showing the influence of crude MC-LR concentration and temperature on crude MC-LR biodegradation ratio, (**e**) Response surface of crude MC-LR concentration and pH on crude MC-LR biodegradation ratio, (**f**) Contour map showing the influence of crude MC-LR concentration and pH on crude MC-LR biodegradation ratio).

To predict the interactive effects of temperature, pH and MC-LR concentration on MC-LR biodegradation efficiency, a three-dimensional (3D) and two-dimensional (2D) response surface and contour plot were applied. The response surface and contour plot analysis could be acquired via plotting the fitted binomial equation, where two environmental factors are altered, while the third environmental factor is held constant at a zero level for 60 min, as displayed in Figure 5.

Figure 5a,b demonstrates the altered interaction of temperature and pH on MC-LR biodegradation efficiency when 3 µg/mL MC-LR is held constant for 60 min. The data indicate that the MC-LR biodegradation efficiency progressively increased as temperature or pH increased but declined at a point. When temperature or pH decreased, the MC-LR biodegradation efficiency also increased but decreased at a point. At a low level of 20 °C and pH 5, the MC-LR biodegradation efficiency was 57.7%. When pH was increased to 9, the toxin’s biodegradation efficiency decreased from 57.7% to 38%. At 30 °C and pH 7, a significant rise in the toxin’s biodegradation efficiency from 38% to 100% was recorded. Interestingly, a fall in the biodegradation efficiency of MC-LR from 100% to 60.3% was attained at 40 °C and pH 5. The observation indicates that the altered effect of temperature and pH on MC-LR biodegradation was similar, making their interaction a significant one.

Figure 5c,d depicts the effect of temperature and MC-LR concentration on MC-LR biodegradation efficiency when pH 7 is held constant for 60 min. The data uncover that the MC-LR biodegradation efficiency steadily increased as the temperature increased or the MC-LR concentration decreased. When temperature decreased or MC-LR concentration increased, the toxin’s biodegradation efficiency also declined. At a low level of 20 °C and 1 µg/mL MC-LR, the toxin’s biodegradation efficiency was 42%. When the MC-LR concentration was increased to 5 µg/mL, the toxin’s biodegradation efficiency decreased from 42% to 30.4%. At 30 °C and 3 µg/mL MC-LR, a notable rise in the toxin’s biodegradation efficiency from 30.4% to 100% was discovered. Nonetheless at 40 °C and 1 µg/mL MC-LR, the toxin’s biodegradation efficiency fell from 100% to 31%. The observed results highlight the influence of temperature and MC-LR concentration, put forth, on the MC-LR biodegradation efficiency.

Figure 5e,f reveals the effect of pH and MC-LR concentration on the MC-LR biodegradation efficiency when 30 °C is held constant for 60 min. The data suggest that MC-LR biodegradation efficiency gradually rose as the pH increased or MC-LR concentration decreased. When the pH decreased or MC-LR concentration increased, the toxin’s biodegradation efficiency also reduced. At a low level of pH 5 and 1 µg/mL MC-LR, the toxin’s biodegradation efficiency was 55%. When the MC-LR concentration was increased to 5 µg/mL, the toxin’s biodegradation efficiency rose from 55% to 63.6%. At pH 7 and 3 µg/mL MC-LR, a remarkable rise in the toxin’s biodegradation efficiency from 63.6% to 100% was noted. On the other hand, at pH 9 and 1 µg/mL MC-LR, the toxin’s biodegradation efficiency dropped from 100% to 46%. The results presented above illustrate the effect that pH and MC-LR concentration exert on the MC-LR biodegradation efficiency. 

The data obtained from the interactive effects of the environmental factors demonstrated that temperature, pH and MC-LR concentration significantly influenced the MC-LR biodegradation efficiency using strain YF1. A similar altered effect of temperature, pH and MC-LR concentration on MC-LR biodegradation efficiency was earlier reported by Guo et al. [30] using a bacterial community. 

## 3. Conclusions

In this study, the biodegrading characteristics of *Sphingopyxis* sp. YF1 under diverse environmental factors were determined and optimized via RSM. Strain YF1 completely biodegraded MC-LR at a maximal rate of 5 µg/mL/h, contributing to the biological treatment of MC-contaminated water bodies. Varying environmental factors, including temperature, pH and MC-LR concentration, were found to play a significant role in the biodegradation rate. Using experimental design expert software, BBD in RSM, the two multinomial mathematical models were established to optimize the biodegradation conditions of MC-LR. Performing a 17-batch experiment with the BBD, 3D and 2D response surface and contour lines models were also established to examine the interactive effect of the multiple environmental factors on MC-LR biodegradation. The ANOVA analysis demonstrated that the model was significant and that the model’s prediction of MC-LR biodegradation was also validated and fit with the experiment. The optimum MC-LR biodegradation conditions were observed at 30 °C, pH 7 and 3 µg/mL MC-LR. Under these conditions, the biodegradation efficiency of MC-LR reached 100% after 60 min. To our knowledge, this is the first time RSM has been utilized to optimize the biodegrading conditions of MC-LR using a bacteria strain. The determination of the optimal environmental factors will aid to unfold the detailed MC-LR biodegrading mechanism by strain YF1 and its practical application to remove MC-LR from the environment.

## 4. Materials and Methods

### 4.1. Materials and Reagents

Methanol and trifluoroacetic acid utilized for high performance liquid chromatography (HPLC) were bought from the Dikma Technology Incorporation (Foothill Ranch, CA, USA). The mineral salt medium (MSM) used for the bacterial culture, acquisition and MC-LR biodegradation contained (g/L) MgSO_4_·7H_2_O 1.0, KH_2_PO_4_ 0.5, K_2_HPO_4_ 4.0, NaCl 1.0, CaCl_2_ 0.02, FeSO_4_ 0.005, MnCl_2_·4H_2_O 0.005, ZnCl_2_ 0.005 and CuCl_2_ 0.0005. The phosphate buffer (PBS) medium contained NaCl, Na_2_HPO_4_·12H_2_O and NaH_2_PO_4_·2H_2_O, 10 mM, pH 7.0. The nutrient broth (NB) medium used for bacteria growth contained 0.5 g beef extract, 0.5 g sodium chloride and 1 g peptone [7]. 

### 4.2. Extraction of MC-LR

The crude MC extract was obtained from algae samples collected from the Dongfang hong fresh-water pond located in Human Province, Changsha City. The algae cells were extracted with 70% methanol and 30% water preceded by centrifugation at 4500 r/min for 15 min. Freezing and thawing of algae cells (thrice), ultrasonication for 20 min and centrifugation at 13,500 r/min for 15 min were carried out, and the pH of the supernatant was adjusted to pH 3.5 and refrigerated at 4 °C overnight. The supernatant was centrifuged at 13,500 r/min for 20 min and left to stand still for at least 5 h, and the pH was re-adjusted to pH 7. The supernatant was again left to stand still for at least 3 h following centrifugation at 13,500 r/min for 20 min. The obtained supernatant was purified using a solid phase extraction device. The column was first activated with 20 mL methanol and water, respectively, and then, 15 mL 20% methanol and 15 mL 30% methanol were used for gradient elution to remove impurities. The obtained algae toxin was evaporated at 65 °C in a rotational vacuum cylinder to remove part of the methanol and water. The purified algae toxin acquired was analyzed using HPLC and HPLC coupled with an ultra-high resolution LTQ Orbitrap Velos Pro ETD mass spectrometry (Thermo Scientific, Dreieich, Hesse, Germany), equipped with an electrospray ionization interface (HPLC-ESI-MS) (LTQ Orbitrap Velos Pro ETD, Thermo Fisher, Waltham, MA, USA). The analytical column used was a Zorbax Extend C_18_ column (4.6 × 150 mm, 5 µm, Agilent, Palo Alto, CA, USA). The crude MC-LR was compared with standard MC-LR (Alexis Corporation (Lausen, Switzerland) by putting into consideration the retention time and the absorbance at 238 nm at the same time. The toxin was stored at −20 °C until the further biodegradation analysis. 

### 4.3. Isolation of Bacterial Strain

Water samples were collected from Lake Taihu and inoculated into 45 mL MSM. A MC-degrading bacterium was obtained after 5 days. Through morphology, 16S rRNA phylogenetic analysis, classification and taxonomy, the isolate YF1 was affiliated with the genus *Sphingopyxis* and closely resembled *Sphingopyxis macrogoltabida,* as previously documented [7]. The bacterium YF1 was stored at −80 °C until the further biodegradation analysis.

### 4.4. Crude MC-LR Biodegradation by Strain YF1

To study the MC-LR biodegrading ability of strain YF1, the bacterial strain was cultured for 72 h in NB medium (pH 7) in 100 mL distilled water at 30 °C, as earlier reported [7]. When the OD_600_ value of strain YF1 was 0.6 using SpectraMaxPlus 384 (Molecular Device, Silicon Valley, CA, USA), bacterial cells were harvested by centrifugation (5000× *g*, 15 min, 4 °C). The supernatant was decanted, and the washing procedure was repeated thrice using 10 mM PBS (pH 7.0) with centrifugation as above. The cells obtained were re-suspended into MSM containing crude MC-LR and cultured under different incubation conditions. The biodegrading ability of strain YF1 was assessed at different temperatures (4 °C, 20 °C, 30 °C or 40 °C), pH (5, 7 or 9) and crude MC-LR concentrations (1, 3 or 5 µg/mL). Controls were prepared in the same way without bacterial cells. An aliquot (50 µL) of samples was withdrawn at intervals of every 15 min for 1 hr. The concentrations of MC-LR in supernatants were detected using HPLC-ESI-MS after centrifugation (12,000× *g*, 15 min, 4 °C). The above biodegradation experiment that assessed the biodegrading performance of YF1 under varying temperatures, pH and crude MC-LR concentrations were carried out in duplicate. 

### 4.5. Analysis of Crude MC-LR

The Agilent 1100 HPLC machine (Agilent, Palo Alto, CA, USA) with a Zorbax Extend C_18_ column (4.6 × 150 mm, 5 µm) and a variable wavelength detector set at 238 nm was employed to analyze the MC-LR concentration. The mobile phase was a mixture of 0.05% trifluoroacetic acid aqueous solution and methanol (37:63, *v*/*v*) set at a flow rate of 0.8 mL/min. The injection volume was 10 µL, and the column temperature was maintained at 40 °C. All the solutions were prepared using distilled water. 

### 4.6. Response Surface Method Design for Optimizing the Environmental Factors

To identify the best condition for MC-LR biodegradation using strain YF1, RSM was applied. RSM is a statistical experimental process optimization tool that uses a polynomial equation to express the interaction or association between operating parameters and an experimental response [25]. BBD was proposed in RSM to evaluate the influence of environmental factors, including temperature (20 °C, 30 °C or 40 °C), pH (5, 7 or 9) and MC-LR concentration (1, 3 or 5 µg/mL), on MC-LR biodegradation using strain YF1. The three main BBD environmental factors (temperature, pH and MC-LR concentration) were denoted by X1, X2 and X3 (Table 1), respectively, and it was performed. Design-Expert (Design-Expert 8.0.6) was used to analyze the experimental data. Furthermore, a 17-run experiment was generated and carried out, including five replications performed at the center point, as illustrated in Table 2. The low, medium and high experimental levels of each independent environmental factor depicted in Table 1 were coded as −1, 0 and +1, respectively. The relationship between the coding value and the real value was defined by the following equation

(1) $$x_{i}=\frac{X_{i}-X_{o}}{\Delta X_{i}}\;$$

where i is the encoding value of the independent variable, X_i_ is the actual test level of the independent variable, X_o_ is the actual value of the horizontal center of the experiment and $\Delta X_{i}$ is a single environmental factor increment. The relationship between the three environmental factors studied (X1, X2 and X3) and their biodegradation efficiency on MC-LR (Y) by strain YF1 was also expressed in a polynomial equation as

(2) $$Y=b_{o}+{\;b}_{i}X1+{\;b}_{j}X2+{\;b}_{k}X3+\ldots$$

where Y is the predicted response (MC-LR biodegradation); X1, X2 and X3 are the independent environmental factors, and b_o_, b_i_, b_j_ and b_k_ (i = 1, 2, 3; j = 1, 2, 3; k = 1, 2, 3) are the regression coefficient. Here, a second order polynomial in the form of a quadratic model was used

(3) $$Y={\;b}_{o}+{\;b}_{i}X1+{\;b}_{j}X2+{\;b}_{k}X3+{\;b}_{\mathrm{ij}}X1X2+{\;b}_{\mathrm{ik}}X1X3+b_{\mathrm{jk}}X2X3+{\;b}_{\mathrm{ii}}\;X1^{2}+{\;b}_{\mathrm{jj}}X2^{2}+{\;b}_{\mathrm{kk}}X3^{2}$$

The properties of the polynomial model equation fitting were expressed by the determination of the correlation coefficient (R^2^), and the statistical significance was verified by the F-value (Fisher variation ratio) or probability value (*p* < 0.05). The optimal biodegradation conditions and the maximum biodegradation efficiency of MC-LR by strain YF1 were predicted by a design expert analysis. 

## Figures and Tables

**Table 1:** Environmental factors, level and coding.

Environmental Factors	Code	Coding Level		
		−1	0	+1
Temperature (°C)	X1	20	30	40
pH	X2	5	7	9
Crude MC-LR concentration (µg/mL)	X3	1	3	5

**Table 2:** Box-Behnken Design scheme for detecting crude MC-LR biodegradation under different environmental factors.

Test Number	Environmental Factor Coding Level			60 Min Biodegradation Rate (%)
	X1	X2	X3	
1	0	−1	−1	55
2	0	0	0	100
3	0	0	0	100
4	+1	−1	0	60.3
5	−1	+1	0	38
6	0	0	0	100
7	+1	0	−1	31
8	0	−1	+1	63.6
9	0	+1	+1	50.8
10	−1	−1	0	57.7
11	+1	0	+1	40
12	+1	+1	0	29.7
13	−1	0	−1	42
14	−1	0	+1	30.4
15	0	0	0	100
16	0	0	0	100
17	0	+1	−1	46

**Table 3:** Analysis of variance table for Box-Behnken response surface model of crude MC-LR biodegradation by *Sphingopyxis* sp. YF1.

Source	Sum of Square	Degree of Freedom	Mean Square	F-Value	*p*-Value Prob > F	
Model	11,996.08	9	1332.90	68.68	<0.0001	significant
A-Temperature	6.30	1	6.30	0.32	0.5866	
B-pH	649.80	1	649.80	33.48	0.0007	
C-MC-LR concentration	14.58	1	14.58	0.75	0.4148	
AB	29.70	1	29.70	1.53	0.2559	
AC	106.09	1	106.09	5.47	0.0520	
BC	3.61	1	3.61	0.19	0.6792	
A^2^	5392.61	1	5392.61	277.88	<0.0001	
B^2^	1332.19	1	1332.19	68.65	<0.0001	
C^2^	3387.08	1	3387.08	174.54	<0.0001	
Residual	135.84	7	19.41			
Lack of Fit	135.84	3	45.28			
Pure Error	0.000	4	0.000			
Cor Total	12,131.92	16				

Std Dev = 4.41, Mean = 61.44, C.V% = 7.17, PRESS = 2173.48, R-Squared = 0.9888, Adj R-Squared = 0.9744, Pred R-Square = 0.8208 and Adeq Precisior = 21.173.

## Data Availability

Not applicable.

## Supplementary Materials

The following supporting information can be downloaded at: https://www.mdpi.com/article/10.3390/toxins14040240/s1, Table S1: Estimated coded factors of the Box-Behnken model of crude MC-LR biodegradation by *Sphingopyxis* sp. YF1 and their confidence interval (CI) and variance inflation factor (VIF)
