# Supplementary material for: Optimization of Biodegradation Characteristics of Sphingopyxis sp. YF1 against Crude Microcystin-LR Using Response Surface Methodology
Source: Toxins (Basel). 2022 Mar 27;14(4):240. doi: 10.3390/toxins14040240 (PMC9026303; doi:10.3390/toxins14040240)
Supplement: Supplementary file 1 [file toxins-14-00240-s001.zip › toxins-1565651-supplementary.pdf]

toxins

MDPI

Optimization of biodegradation characteristics of *Sphingopyxis* sp. YF1 against crude microcystin-LR using response surface methodology

Isaac Yaw Massey, Tangjian Peng, Cai Danping and Fei Yang

Table S1. Estimated coded factors of the Box-Behnken model of crude MC-LR biodegradation by *Sphingopyxis* sp. YF1 and their confidence interval (CI) and variance inflation factor (VIF)

Factor	Coefficient Estimate	Degree of freedom	Standard error	95% CI Low	95% CI High	VIF
Intercept	100.00	1	1.97	95.34	104.66	
A-Temperature	-0.89	1	1.56	-4.57	2.80	1.00
B-pH	-9.01	1	1.56	-12.70	-5.33	1.00
C-MC-LR concentration	1.35	1	1.56	-2.33	5.03	1.00
AB	-2.73	1	2.20	-7.93	2.48	1.00
AC	5.15	1	2.20	-0.058	10.36	1.00
BC	-0.95	1	2.20	-6.16	4.26	1.00
A^2	-35.79	1	2.15	-40.86	-30.71	1.01
B^2	-17.79	1	2.15	-22.86	-12.71	1.01
C^2	-28.36	1	2.15	-33.44	-23.29	1.01
